# WHAT INTERFERES WITH OLDER ADULTS’ PARTICIPATION IN A MODIFIED PRESCRIBED EXERCISE PROGRAM?

**DOI:** 10.1093/geroni/igad104.3695

**Published:** 2023-12-21

**Authors:** Justine Sefcik, Sean Harrison, Marjorie Skubic, Therese Richmond, Nancy Hodgson, George Demiris

**Affiliations:** Drexel University College of Nursing and Health Professions, Philadelphia, Pennsylvania, United States; University of Pennsylvania, Philadelphia, Pennsylvania, United States; University of Missouri, Columbia, Missouri, United States; University of Pennsylvania, Philadelphia, Pennsylvania, United States; University of Pennsylvania, Philadelphia, Pennsylvania, United States; University of Pennsylvania, Philadelphia, Pennsylvania, United States

## Abstract

Exercise programs have great potential to help keep older adults active and independent in their homes. However, barriers to participation in a virtual coaching exercise program aimed at fall prevention is not well understood. This qualitative descriptive study with a conventional content analysis examined older adults’ perceptions of what interfered with their participation in a modified 3-month Otago exercise program. Ten older adults participated in five bi-weekly telephone coaching calls and interviews where they were asked about their last two weeks of exercise participation. Participants were mean age 71.3 (range 66 - 81), and mostly female (90%), Black (70%), and non-Hispanic (90%). While most of the participants routinely participated in their prescribed exercises, a few had difficulty completing the plan for some weeks. We identified two themes of what participants expressed had gotten in their way of routinely exercising: 1) distracted and busy (e.g., prepping for the holidays, death in the family, needing to clean home) and 2) physical problems (e.g., pain, being sick, injury). Only one participant said they lacked motivation, which resulted in them exercising less than their initial intention. These findings suggest that older adults want to participate in a routine exercise program, however, they sometimes have other priorities or physically cannot exercise. Program implementers should consider expanding timelines of physical training to account for weeks when older adults are unable to exercise and provide modifications for participants when ill, experiencing chronic pain, or have a new injury.

